# Supplementary material for: Dielectric Polarization and Electrorheological Response of Poly(ethylaniline)-Coated Reduced Graphene Oxide Nanoflakes with Different Reduction Degrees
Source: Polymers (Basel). 2020 Oct 29;12(11):2528. doi: 10.3390/polym12112528 (PMC7692228; doi:10.3390/polym12112528)
Supplement: Supplementary file 1 [file polymers-12-02528-s001.docx]

Supplementary Information

**Dielectric Polarization and Electrorheological Response of Poly(ethylaniline)-Coated Reduced Graphene Oxide Nanoflakes With Different Reduction Degrees**

Yudong Wang, Min Yang, Honggang Chen, Xiaopeng Zhao, Jianbo Yin

Smart Materials Laboratory, Department of Applied Physics, Northwestern Polytechnical University, Xi’an 710129, P. R. China.

*Corresponding author: E-mail: [jbyin@nwpu.edu.cn](mailto:jbyin@nwpu.edu.cn)


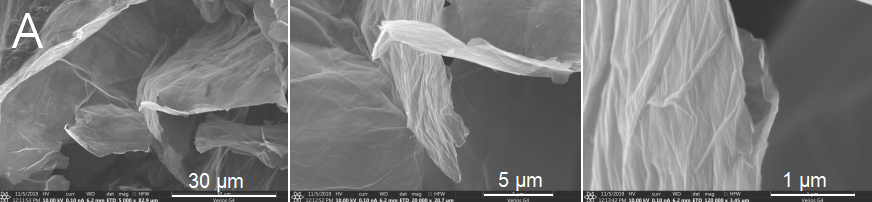

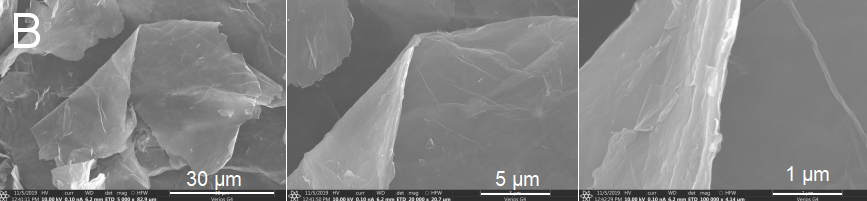

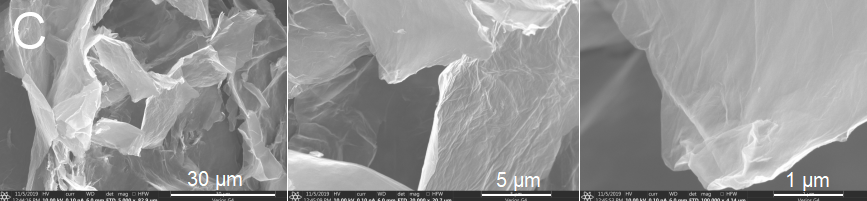

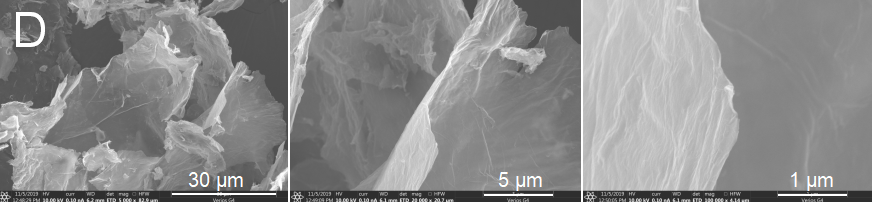

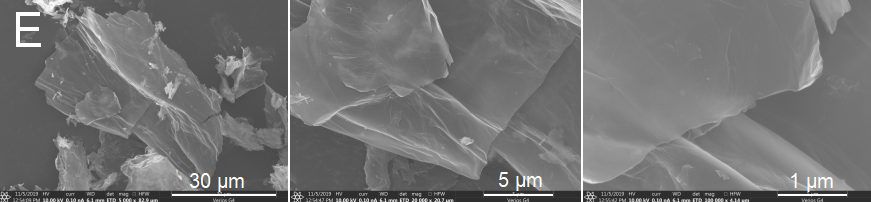


**Figure S1.** SEM images of pure GO (**A**) and rGO obtained by reduction with hydrazine of 25 μL (**B**), 50 μL (C), 150 μL (D) and 400 μL (E).

**Figure S2.** (**A**) XPS full spectra of as-synthesized PEANI/GO (a) and PEANI/rGO obtained by reduction with different hydrazine of 70 μL (b), 140 μL (c), 420 μL (d), and1400 μL (e); (**B**) XPS full spectra of pure GO (a) and rGO obtained by reduction with hydrazine of 25 μL (b), 50 μL (c), 150 μL (d), and 400 μL (e).

**Table S1.** Conductivity of pure rGO obtained by reduction with different amounts of hydrazine at 25 ℃.

| **hydrazine**  **(μL)** | 25 | 50 | 150 | 400 |
| --- | --- | --- | --- | --- |
| *σ* (S/m) | 40 ± 3 | 55 ± 7 | 85 ± 10 | 138 ± 11 |

**Table S2.** Comparison of the relaxation times of calculated interfacial polarization (*λ*_MW_) and measured electric polarization (*λ*_1_) for PEANI shell in ERFs of PEANI/rGO obtained by reduction with different amounts of hydrazine at 25 ℃.

| **hydrazine**  **(μL)** | ***ε*_p1_** | ***σ*_1_ (S/m)** | ***λ*_MW1_** | ***λ*_1_** |
| --- | --- | --- | --- | --- |
| 70 | 5.60 | 1.09 × 10^-7^ | 9.26 × 10^-4^ | 1.36 × 10^-4^ |
| 140 | 4.78 | 9.02 × 10^-8^ | 1.04 × 10^-3^ | 4.50 × 10^-4^ |
| 420 | 3.75 | 5.11 × 10^-8^ | 1.66 × 10^-3^ | 1.50 × 10^-3^ |
| 1400 | 3.19 | 2.56 × 10^-8^ | 3.11 × 10^-3^ | 2.30 × 10^-3^ |

The Maxwell-Wagner interfacial polarization formula below was used to estimate the relaxation times according to the conductivity (*σ*_p_) of PEANI.

$\lambda_{\mathrm{MW}}=\varepsilon_{0}\frac{2\varepsilon_{f}^{'}+\varepsilon_{p}^{'}-\phi(\varepsilon_{p}^{'}-\varepsilon_{f}^{'})}{2\sigma_{f}+\sigma_{p}-\phi(\sigma_{p}-\sigma_{f})}$ (1)

where, 𝜀_0_ is the permittivity of vacuum, *ε*′_p_ calculated by mixture rule lg*ε*′*_∞_*=*ϕ*lg*ε*′_p_+(1-*ϕ)*lg*ε*′_f_ is the real part of permittivity of PEANI shell, *ε*′*_∞_* is the real part of permittivity of total ERFs at high frequency, *ε*′_f_ is real part of permittivity of carrier liquid (*ε*′_f_ =2.71), *σ*_f_ is the DC conductivity of carrier liquid which can be negligible during calculation (~10^-15^ S/cm), and *ϕ* = 4.5 % is the particle volume fraction. Table S2 lists the data of the relaxation times. It is seen that the predicted value of *λ*_MW_ calculated by Eq. 1 is close to that of *λ* measured in dielectric spectra. So, it can be clarified that the dielectric relaxation processes shown in Fig. 5 originate from the interfacial polarizations of PEANI shell. Because it was difficult to get the real conductivity of rGO core, we here did not calculate the $\lambda_{\mathrm{MW}}$ of rGO core.

**Figure S3.** Temperature dependence of permittivity spectra of ERFs of PEANI/rGO obtained by reduction with different amounts of hydrazine: (**A**) 70 μL, (**B**) 140 μL, (**C**) 420 μL and (**D**) 1400 μL.

**Figure S4.** Temperature dependence of the reciprocal of $\lambda$for PEANI shell and rGO core in PEANI/rGO obtained by reduction with different amounts of hydrazine.
